# Quantitative analysis of basic fibroblast growth factor and vascular endothelial growth factor in human colorectal cancer.

**DOI:** 10.1038/bjc.1998.575

**Published:** 1998-09

**Authors:** M. Landriscina, A. Cassano, C. Ratto, R. Longo, M. Ippoliti, B. Palazzotti, F. Crucitti, C. Barone

**Affiliations:** Institute of Internal Medicine and Geriatrics, Medical Oncology Section, Catholic University, Rome, Italy.

## Abstract

Tumour growth is angiogenesis dependent. Some authors suggest a prognostic role of microvessel count in colorectal cancer. We tested the role of basic fibroblast growth factor (bFGF) and vascular endothelial growth factor (VEGF) in the switch to the angiogenic phenotype in 35 patients with colorectal cancer at different stages of disease. We evaluated the two angiogenic factors, by enzyme-linked immunosorbent assay (ELISA), in tumour, peritumoral mucosa, pathological mesenteric and peripheral blood. We used ten endoscopic intestinal biopsies and ten peripheral blood samples from healthy subjects as control. bFGF was significantly lower in tumour tissues and in peritumoral mucosas than in healthy mucosas, whereas VEGF was up-regulated in tumours but not in peritumoral mucosa. Both angiogenic factors were greatly increased in mesenteric blood. VEGF tumour and serum levels were significantly correlated with the stage of disease. bFGF tumour and serum concentration were not correlated with the stage of disease. The high levels of bFGF in mesenteric blood suggest that this growth factor might be abnormally released from tumour tissue and peritumoral mucosa and could function as an early effector in the switch to the angiogenic phenotype. In contrast, VEGF, whose levels show a significant correlation with the stage of disease, could act in a following step, supporting tumour progression.


British Journal of Cancer (1998) **78**(6), 765–770
© 1998 Cancer Research Campaign

### Quantitative analysis of basic fibroblast growth factor and vascular endothelial growth factor in human colorectal cancer

**M Landriscina[1], A Cassano[1], C Ratto[2], R Longo[1], M Ippoliti[2], B Palazzotti[3], F Crucitti[2] and C Barone[1]**

[1]Institute of Internal Medicine and Geriatrics, Medical Oncology Section, [2]Institute of Clinical Surgery and [3]Institute of General Pathology, Catholic University, Largo F Vito, 1-00168 Rome, Italy

**Summary** Tumour growth is angiogenesis dependent. Some authors suggest a prognostic role of microvessel count in colorectal cancer. We tested the role of basic fibroblast growth factor (bFGF) and vascular endothelial growth factor (VEGF) in the switch to the angiogenic phenotype in 35 patients with colorectal cancer at different stages of disease. We evaluated the two angiogenic factors, by enzyme-linked immunosorbent assay (ELISA), in tumour, peritumoral mucosa, pathological mesenteric and peripheral blood. We used ten endoscopic intestinal biopsies and ten peripheral blood samples from healthy subjects as control. bFGF was significantly lower in tumour tissues and in peritumoral mucosas than in healthy mucosas, whereas VEGF was up-regulated in tumours but not in peritumoral mucosa. Both angiogenic factors were greatly increased in mesenteric blood. VEGF tumour and serum levels were significantly correlated with the stage of disease. bFGF tumour and serum concentration were not correlated with the stage of disease. The high levels of bFGF in mesenteric blood suggest that this growth factor might be abnormally released from tumour tissue and peritumoral mucosa and could function as an early effector in the switch to the angiogenic phenotype. In contrast, VEGF, whose levels show a significant correlation with the stage of disease, could act in a following step, supporting tumour progression.

**Keywords:** basic fibroblast growth factor; vascular endothelial growth factor; colorectal cancer; angiogenic factor; enzyme-linked immunosorbent assay

Angiogenesis consists of the sprouting of capillaries from pre-existing vessels. This physiological process is fundamental in reproduction, development and wound repair (Folkman, 1995). Angiogenesis is deregulated in several pathological conditions. Growth of tumour mass is made possible not only because of perfusion of blood through the tumour, but also because of the paracrine stimulation of tumour cells by numerous growth factors and matrix proteins that are produced by the new capillary endothelium. It has been suggested that the switch to the angiogenic phenotype depends on a net balance between positive and negative angiogenic factors released by the tumour (Folkman, 1995).

Weidner et al (1991) demonstrated a statistically significant correlation between the incidence of metastases and intratumoral microvessel density (IMD) in invasive breast carcinomas. This finding was confirmed in several studies in early-stage breast cancer (Weidner et al, 1992), melanoma (Graham et al, 1994), cervical (Smith-McCune and Weidner, 1994) and prostate carcinoma (Weidner et al, 1993).

Vermeulen et al (1995) described increased IMD in areas of invasive colon cancer compared with IMD in areas of in situ growth without any prognostic evaluation. Moreover, IMD was found to be greater in metastatic tumour than in non-metastatic tumour (Takahashi et al, 1995). The prognostic role of microvessel count in colorectal cancer was investigated by several authors with controversial results. IMD was significantly correlated with the presence of liver metastasis (Tomisaki et al, 1996), haematogenous metastasis (Tanigawa et al, 1997), relapse and overall survival (Frank et al, 1995; Takahashi et al, 1997). Opposite results are reported by Bossi et al (1995), who did not find any association among IMD, metastasis, stage of disease and patient survival, and by Lindmark et al (1996) who demonstrated a significant correlation between increased IMD and better survival. All these different data could be due to different methods used to process tissues and to quantify microvessels.

Basic fibroblast growth factor (bFGF) has a potent mitogenic activity for a wide variety of mesoderm–neuroectoderm-derived cells. This peptide stimulates vascular endothelial cell proliferation and virtually all these cells either produce or have receptors for bFGF. It has been demonstrated that bFGF is involved in the neoplastic angiogenesis of several types of tumours (melanomas, glioblastoma, Kaposi sarcoma, pancreas, renal, breast and lung tumours) (Basilico and Moscatelli, 1992).

Recently, considerable interest has developed in the possible participation of the endothelial mitogen vascular endothelial growth factor (VEGF) in malignant tumour growth. VEGF is mitogenic for a variety of large- and small-vessel endothelial cells, induces the production of tissue factors, collagenase, plasminogen activators and their inhibitors, and stimulates hexose transport in these cells as well. VEGF is also known as 'vascular permeability factor' by virtue of its permeability enhancing effects. VEGF expression has been demonstrated in several human cancer cell lines in vitro and in surgically resected tumours of the gastro-intestinal tract, ovary, brain, breast and kidney (Thomas, 1996).

*Received 30 October 1997*
*Revised 16 February 1998*
*Accepted 3 March 1998*

*Correspondence to:* C Barone, Università Cattolica del S. Cuore, Istituto di Medicina Interna e Geriatria; Sezione di Oncologia Medica, Largo A Gemelli, 8-00168 Rome, Italy

**Table 1** Summary of analysed samples

| Tissues analysed | No. of samples |
|---|---|
| No. of patients | 35 |
| bFGF dosage | |
| Tumour | 32 |
| Peritumoral mucosa 1 cm | 10 |
| Peritumoral mucosa 5 cm | 32 |
| Peritumoral mucosa 10 cm | 10 |
| Post-operative mucosa | 7 |
| Healthy mucosa | 10 |
| Pathological mesenteric blood | 20 |
| Pathological peripheral blood | 21 |
| Healthy peripheral blood | 10 |
| VEGF dosage | |
| Tumour | 20 |
| Peritumoral mucosa | 20 |
| Healthy mucosa | 10 |
| Pathological mesenteric blood | 20 |
| Pathological peripheral blood | 20 |
| Healthy peripheral blood | 10 |

VEGF and bFGF are both secreted, although some authors have demonstrated that bFGF is secreted by an alternative secretion pathway (Bussolino et al, 1996). Moreover, recent studies demonstrated that VEGF receptor is not specific for endothelial cells, as previously reported for bFGF receptor (Brown et al, 1997; Ergun et al, 1997).

In an effort to better understand the role of bFGF and VEGF in colorectal cancer, we measured by enzyme-linked immunosorbent assay (ELISA) the levels of these angiogenic factors in a series of human colorectal cancer and in blood samples from peripheral and mesenteric veins.

### PATIENTS AND METHODS

### Patients

Between February and December 1996, specimens were obtained from 35 patients with colorectal adenocarcinoma. The group was composed of 20 men and 15 women. The mean age was 62.7 years (range 32–82). Primitive tumour was present in the right colon in 12 cases, in the transverse colon in two cases, in the left colon in five cases, in the sigmoid colon in 11 cases and in the rectum in five cases. All cases were staged according to Dukes' (modified according to Astler-Coller) and TNM classifications; Dukes: $B_1$, three cases; $B_2$, ten cases; $C_1$, four cases; $C_2$, ten cases; D, eight cases; TNM: $T_2$, eight cases; $T_3$, 24 cases; $T_4$, three cases; node negative, 17 cases; node positive, 18 cases; metastasis negative, 27 cases; metastasis positive, eight cases (De Vita et al, 1997). Histological grading was $G_2$ in 19 cases and $G_3$ in 16 cases.

Thirty-two specimens of tumours and healthy mucosa at 5 cm from the tumour were collected; moreover, in ten patients, specimens of healthy mucosa at 1 and 10 cm from the tumour were also collected. Surgical procedures allowed us to collect blood samples from mesenteric vein in only 20 patients; 21 blood specimens from the brachial vein (peripheral blood) were collected in the same group of patients. All these samples were intraoperative specimens, obtained during the surgical removal of the tumour. Seven patients were submitted to an endoscopic examination with a biopsy of colon mucosa 6 months after surgery. A control group of informed and voluntary subjects was selected in the same range of age and sex as the pathological one. Among these subjects we collected ten endoscopic specimens of bowel mucosa and ten peripheral blood samples.

The tissues were divided in 125-$mm^3$ pieces, immediately frozen in liquid nitrogen and stored at –80°. Samples were thawed only once and were analysed 15–30 days after collection; one piece was used for histology, one each for bFGF and VEGF ELISAs. Histology was confirmed in all cases. Serum was obtained from blood specimens and stored at –80°.

### Samples

Tissue specimens were homogenized in potassium phosphate buffer (200 mg wet weight $ml^{-1}$) as described previously in Landriscina et al (1996), protein concentration was determined spectrophotometrically with a Biorad kit according to the manufacturer's procedures. Blood sera were analysed directly.

**Table 2** Mean bFGF and VEGF content in tumour, mucosa and blood

| Tissue | bFGF levels | | VEGF levels | |
|---|---|---|---|---|
| | No. of patients | bFGF (pg $mg^{-1}$ proteins)[a] | No. of patients | VEGF (ng $mg^{-1}$ proteins)[b] |
| Tumour | 32 | 1066.4 ± 514 | 20 | 14.3 ± 7 |
| Peritumoral mucosa (5 cm) | 32 | 1954.2 ± 692 | 20 | 5.3 ± 2 |
| Healthy mucosa | 10 | 3694.0 ± 1501 | 10 | 4.7 ± 2 |
| **Tissue** | **bFGF levels** | | **VEGF levels** | |
| | No. of patients | bFGF (pg $ml^{-1}$ serum)[c] | No. of patients | VEGF (ng $ml^{-1}$ serum)[d] |
| Pathological mesenteric blood | 20 | 58.3 ± 37 | 20 | 15.0 ± 8 |
| Pathological peripheral blood | 21 | 14.3 ± 12 | 20 | 11.2 ± 4 |
| Healthy peripheral blood | 10 | 6.1 ± 3 | 10 | 11.8 ± 5 |

[a]Tumour vs peritumoral mucosa $P < 0.0000003$, peritumoral vs. healthy mucosa $P < 0.000009$, tumour vs healthy mucosa $P < 0.0000000002$. [b]Tumour vs peritumoral mucosa $P < 0.000003$, peritumoral vs healthy mucosa NS, tumour vs healthy mucosa $P < 0.0002$. [c]Mesenteric vs peripheral blood $P < 0.000009$, peripheral vs healthy blood $P < 0.04$. [d]Mesenteric vs peripheral blood NS, peripheral vs healthy blood NS.

 © Cancer Research Campaign 1998

**Figure 1** bFGF levels in healthy and pathological tissues (**A** and **B**) and VEGF levels in healthy and pathological tissues (**C** and **D**)

### bFGF dosage bFGF ELISA kit was purchased from Wako Chemical and the procedures included were followed. The kit used three monoclonal antibodies: clones no. 52 and 98 with similar antigenic recognition and clone no. 3H3 HRP-conjugated FAB′ prepared from a monoclonal antibody having another epitope recognition. bFGF was quantified by using a standard curve made by human bFGF ranging from 3 pg ml$^{-1}$ to 500 pg ml$^{-1}$. The chromogenic reaction was read at the absorbance of 490 nm (Watanabe et al, 1991).

### VEGF dosage

A competitive enzyme immunoassay that measures natural and recombinant forms of VEGF was purchased from Chemicon International and the procedures included were followed. The kit used a polyclonal anti-VEGF antibody. VEGF concentration was dosed according to a standard curve ranging from 0.19 to 800 ng ml$^{-1}$. The chromogenic reaction was read at 490 nm.

### Statistical analyses

Differences in mean content of bFGF and VEGF among tumour, mucosa and blood samples were analysed by Student's *t*-test. The correlation between bFGF, VEGF and the stage of disease was examined by the Kendall Tau test. For all statistical analyses, the level of significance was set at $P < 0.05$. Statworks statistical software (Statistic for Windows, Statsoft, 1993) was used for all analyses.

### RESULTS

### bFGF levels

We evaluated the bFGF content in 32 specimens of colorectal cancer and in 32 specimens of histologically not infiltrated peritumoral mucosa (5 cm from the tumour); we also tested 20 samples of mesenteric blood and 21 samples of peripheral blood. We used ten endoscopic intestinal biopsies and ten samples of peripheral blood from healthy subjects as controls (Table 1). The analytical patients' data are reported in Table 2 and Figure 1 A and B. bFGF mean content in healthy normal mucosa was 3694.0 ± 1501 pg mg$^{-1}$ of total proteins, in tumour tissues it was 1066.4 ± 514 pg mg$^{-1}$ of total proteins and in peritumoral mucosa it was 1954.2 ± 692 pg mg$^{-1}$ of total proteins. *t*-test demonstrated a highly significant difference between healthy mucosa and tumour tissue ($P < 0.0000000002$), between peritumoral mucosa and

© Cancer Research Campaign 1998

Table 3 Mean bFGF content in mucosa at different distances from tumour and in post-operative mucosa (in 10 of 32 patients)

| Tissue | No. of cases | bFGF (pg $mg^{-1}$ of total proteins) |
|---|---|---|
| Tumour | 10 | 1468.7 ± 447 |
| Peritumoral mucosa I (1 cm) | 10 | 1748.5 ± 525 |
| Peritumoral mucosa II (5 cm) | 10 | 2449.8 ± 417 |
| Peritumoral mucosa III (10 cm) | 10 | 2509.8 ± 604 |
| Intraoperative mucosa | 7 | 1679.0 ± 903 |
| Postoperative mucosa (6 months) | 7 | 2294.8 ± 612 |

Table 4 Correlation between VEGF levels and stage of disease

| | *P*-values (determined by the Kendal Tau test) | | |
|---|---|---|---|
| | Tumour | Mesenteric blood | Peripheral blood |
| TNM | 0.02 | 0.007 | 0.002 |
| $T_{1-4}$[a] | 0.009 | 0.002 | 0.002 |
| N–/N+[a] | NS | NS | NS |
| M–/M+[a] | 0.004 | NS | 0.01 |
| Dukes | 0.02 | 0.005 | 0.03 |

[a]T, primitive tumour; N, node metastasis; M, distant metastasis.

tumour tissue ($P < 0.0000003$) and between peritumoral mucosa and healthy mucosa ($P < 0.000009$).

Mean bFGF level in healthy peripheral blood was 6.1 ± 3 pg $ml^{-1}$, in pathological peripheral blood it was 14.3 ± 12 pg $ml^{-1}$ and in pathological mesenteric blood it was 58.3 ± 37 pg $ml^{-1}$ (Table 2). The difference between pathological mesenteric and pathological peripheral blood was highly significant ($P < 0.000009$). The difference between pathological and healthy peripheral blood was also significant, although to a lesser extent ($P < 0.04$).

In order to explore the role of tumour tissue on bFGF content of peritumoral mucosa, we dosed bFGF at different distances from the primitive tumour and in post-operative endoscopic biopsies from patients who had previously undergone to radical surgery for adenocarcinoma (Table 3). We observed that the bFGF content in mucosa close to the tumour was nearly the same as tumour itself, as expected (1748.5 ± 525 vs 1468.7 ± 447; $P < 0.3$); otherwise bFGF content in mucosa at 5 cm and 10 cm from the tumour was significantly higher than the tumour itself (2449.8 ± 417 and 2509.8 ± 604; $P < 0.00008$ and $P < 0.0004$ respectively). bFGF content in mucosa at 10 cm from the tumour was still significantly lower than the healthy control (2509.8 ± 604 vs 3694.0 ± 1501; $P < 0.04$). Seven patients were submitted to endoscopy 6 months after radical surgery: mean bFGF content of mucosa was higher in post-operative specimens than in intraoperative ones, but this difference was not statistically significant (2294.8 ± 612 vs 1679.0 ± 903; $P < 0.2$). The bFGF content of endoscopic post-operative mucosa was found to be still lower than the mucosa of healthy patients ($P < 0.04$).

### VEGF levels

VEGF levels were determined in 20 adenocarcinomas of the colon–rectum, in 20 peritumoral mucosas (5 cm from the tumour) and in ten endoscopic biopsies from healthy patients (Table 1). Table 2 and Figure 1 C–D report analytical data. Significantly higher mean VEGF levels were observed in tumour tissue (14.3 ± 7 ng $mg^{-1}$ of total proteins) than in peritumoral mucosa (5.3 ± 2; $P < 0.000003$); the difference in VEGF content between tumour and healthy mucosa was also statistically significant (4.7 ± 2; $P < 0.0002$). Apart from bFGF, peritumoral mucosa and healthy mucosa have the same VEGF content ($P < 0.5$). In the same group of patients the mean VEGF levels in mesenteric and peripheral blood were 15.0 ± 8 and 11.2 ± 4 ng $ml^{-1}$ respectively. Normal peripheral blood mean VEGF content was 11.8 ± 5 ng $ml^{-1}$ (Table 2). The difference between pathological VEGF mesenteric and peripheral levels was not statistically significant, nor was the difference between normal and pathological peripheral VEGF content.

### Correlation between VEGF and bFGF levels and stage of disease

The Kendall Tau test demonstrated a statistically significant correlation between VEGF levels and stage of disease (Table 4). We observed a significant correlation between VEGF content in tumour, mesenteric and peripheral blood and Dukes' stage of disease ($P < 0.02$; $P < 0.005$; $P < 0.03$). same correlation was also significant with TNM stage ($P < 0.02$, $P < 0.007$, $P < 0.002$). The correlation was more significant when we compared VEGF levels in tumour, mesenteric and peripheral blood and T evaluated according to the TNM classification ($P < 0.009$; $P < 0.002$; $P < 0.002$). Tumour and peripheral VEGF levels were also correlated with the presence of distant metastases ($P < 0.004$; $P < 0.01$). We did not observe any significant correlation between VEGF content and node metastases according to TNM.

We did not find any significant correlation between tissue or serum bFGF levels and Dukes' or TNM stages, grading as well as node or distant metastases. Moreover, the Kendall Tau test did not demonstrate any significant correlation between bFGF and VEGF levels in tumours, mesenteric and peripheral blood.

### DISCUSSION

The aim of the present study was to investigate the possible regulatory activity of bFGF- and VEGF-driven angiogenesis in colorectal cancer. The experimental model includes colorectal tumours at different stages of disease, with or without node and distant metastases. The content of the angiogenic factors was comparatively evaluated in tumours, peritumoral mucosa that was not infiltrated and mucosa from healthy subjects. In a subgroup of patients we also obtained intraoperative blood samples from the mesenteric vein, which drains blood from the bowel and from the brachial vein (peripheral blood).

We observed that the bFGF content was significantly lower in tumour tissue than in peritumoral mucosa and that the bFGF levels in peritumoral mucosa were unexpectedly lower than in healthy mucosa. To establish that the difference between peritumoral and healthy mucosa did not depend on the method of sampling (intraoperative vs endoscopic), we also measured bFGF in preoperative endoscopic biopsies of peritumoral mucosa in three patients, obtaining values similar to those observed in intraoperative peritumoral mucosa (data not shown). In spite of the low bFGF levels in tumour tissue, its content in mesenteric blood was higher than in the peripheral blood of the same patients. Peripheral blood bFGF levels were still higher than those in healthy subjects.

Even though bFGF lacks a leader sequence for secretion, several reports suggest that bFGF is secreted from bFGF-producing cells

 © Cancer Research Campaign 1998

by an alternative secretion pathway (Bussolino et al, 1996) and that it accumulates in the extracellular matrix (ECM), where it can be released by ECM-degrading enzymes (Vlodavski et al, 1991). In order to explore the possibility that tumour could influence the content of bFGF in tumour itself and in peritumoral mucosa, we measured bFGF levels in the mucosa at different distances from the tumour. We observed that bFGF content in mucosa 5 and 10 cm from the tumour was higher than that of mucosa 1 cm from tumour. Although these data do not investigate the mechanisms of the possible release of bFGF from tumour cells and peritumoral mucosa, they suggest that the presence of tumour could influence bFGF release. Kandel et al (1991) described a similar observation in the multistep development of fibrosarcoma in a transgenic mouse model. They found a change in the localization of bFGF from its normal cell-associated form in the normal dermal fibroblast and mild fibromatosis to extracellular release in aggressive fibromatosis and fibrosarcoma.

These data do not exclude the possibility that the decrease in bFGF might represent an early onset sign of transformation, apart from the presence of the tumour. In support of this possibility, bFGF content in endoscopic biopsies of intestinal mucosa, obtained from patients previously submitted to radical surgery for colorectal cancer, was still lower than in healthy mucosa.

Experimental data demonstrated that the expression of VEGF was up-regulated by an abnormality in p53, which is one of the most commonly mutated tumour-suppressor genes in colorectal cancer (Kieser et al, 1994; Mukhopadhyay et al, 1995; Kang et al, 1997). Takahashi et al (1995) demonstrated that the expression of VEGF and its receptors KDR was higher in metastatic than in non-metastatic neoplasms and that it was directly correlated with the extent of neovascularization and the degree of proliferation. Moreover, VEGF mRNA was found to be ubiquitously expressed in human liver metastases from primary colon or rectal cancers (Warren et al, 1995). Recently Dirix et al (1996) dosed bFGF and VEGF in sera from patients bearing advanced colorectal cancer. They observed that the levels of these angiogenic factors are predictive of the progression of disease. In agreement with these results, Takahashi et al (1997) demonstrated, in patients with node-negative colon cancer, that VEGF expression in tumours, as well as IMD, are significantly related to the recurrence.

We measured VEGF in 20 patients and we found that it was increased in tumour tissue in comparison with peritumoral mucosa as well as with healthy mucosa. Moreover, VEGF levels in mesenteric blood were higher than in peripheral pathological and healthy blood. Statistical analyses demonstrated that VEGF levels in tumour, mesenteric and peripheral blood were significantly correlated with tumour stage according to Dukes' and TNM classifications. We have also compared VEGF levels with the different parameters of TNM. We observed a strong correlation between VEGF and the extent of intestinal wall invasion and a less significant correlation with distant metastases. On the other hand, we did not find any correlation between VEGF levels and the presence of node metastases. These observations suggest that node metastases might not be dependent on VEGF-induced angiogenesis.

The different bFGF and VEGF content between pathological peripheral and mesenteric blood could be due to the short half-life of the two growth factors and to their deposition in solid organs such as kidney, liver and spleen (Edelman et al, 1993; Folkman, 1995).

Folkman (1995) hypothesized that tumour cells, endothelial cells or macrophages may secrete specific growth factors (tumour angiogenesis factors) for the proliferation of endothelial cells, which may stimulate the proliferation of new blood vessels in an expanding tumour volume. The role of these angiogenic proteins on the biological system of colorectal cancer is still not completely understood.

Our data do not characterize the cells involved in the production of bFGF and VEGF, but show that they are probably involved in colorectal cancer angiogenesis with different mechanisms. The lack of any correlation between bFGF levels in tumour and mesenteric blood and the stage of disease could suggest that the release of bFGF from tumour and peritumoral cells is an early but probably not specific event in the switch to the angiogenic phenotype in colorectal cancer. The increased production of VEGF according to the stage and the depth of intestinal wall invasion suggests that it could be involved in a later step of the angiogenesis process. Their sequential secretion could represent one of the possible sequences of events that contribute to neoplastic progression and metastasis. This hypothesis might be in agreement with recent studies that have demonstrated the possible synergistic effects of VEGF and bFGF on the induction of angiogenesis either in vitro (Goto et al, 1993) or in vivo (Asahara et al, 1996).

### ACKNOWLEDGEMENT

The authors are immensely thankful to Professor T Galeotti for his support and encouragement.

### REFERENCES

Asahara T, Bauters C, Zheng LP, Takeshita S, Bunting S, Ferrara N, Symes JF and Isner JM (1995) Synergistic effect of vascular endothelial growth factor and basic fibroblast growth factor on angiogenesis in vivo. *Circulation* **92**: II365–371

Basilico C and Moscatelli D (1992) The FGF family of growth factors and oncogenes. *Adv Cancer Res* **59**: 115–165

Bossi P, Viale G, Lee AK, Alfano R, Coggi G and Bosari S (1995) Angiogenesis in colorectal tumors: microvessel quantification in adenomas and carcinomas with clinicopathological correlations. *Cancer Res* **55**: 5049–5053

Brown LF, Detmar M, Tognazzi K, Abu-Jawdeh G and Iruela-Arispe ML (1997) Uterine smooth muscle cells express functional receptors (flt and KDR) for vascular permeability factor/vascular endothelial growth factor. *Lab Invest* **76**: 245–255

Bussolino F, Albini A, Camussi G, Presta M, Viglietto G, Ziche M and Persico G (1996) Role of soluble mediators in angiogenesis. *Eur J Cancer* **32A**: 2401–2412

De Vita Jr VT, Hellman S and Rosemberg SA (1997) *Cancer – Principles & Practice of Oncology*. JB Lippincott: Philadelphia

Dirix LY, Vermeulen PB, Hubens G, Benoy I, Martin M, De Pooter C and Van Oosterom AT (1996) Serum basic fibroblast growth factor and vascular endothelial growth factor and tumour growth kinetics in advanced colorectal cancer. *Ann Oncol* **7**: 843–846

Edelman ER, Nugent MA and Karnovsky MJ (1993) Perivascular and intravenous administration of basic fibroblast growth factor: vascular and solid organ deposition. *Proc Natl Acad Sci USA* **90**: 1513–1517

Ergun S, Kilic N, Fiedler W and Mukhopadhyay AK (1997) Vascular endothelial growth factor and its receptors in normal human testicular tissue. *Mol Cell Endocrinol* **131**: 9–20

Folkman J (1995) Angiogenesis in cancer, vascular, rheumatoid and other disease. *Nat Med* **1**(1): 27–31

Frank RE, Saclarides TJ, Leurgans S, Speziale NJ, Drab EA and Rubin DB (1995) Tumor angiogenesis as a predictor of recurrence and survival in patients with node-negative colon cancer. *Ann Surg* **222**: 695–699

Goto F, Goto K, Weindel K and Folkman J (1993) Synergistic effects of vascular endothelial growth factor and basic fibroblast growth factor on the proliferation and cord formation of bovine capillary endothelial cells within collagen gels. *Lab Invest* **69**: 508–517

Graham CH, Rivers J, Kerbel RS, Stankiewicz KS and White WL (1994) Extent of vascularization as a prognostic indicator in thin (<0.76 mm) malignant melanomas. *Am J Pathol* **145**: 510–514

© Cancer Research Campaign 1998

Kandel J, Bossy-Wetzel E, Radvanyi F, Klagsbrun M, Folkman J and Hanahan D (1991) Neovascularization is associated with a switch to the export of bFGF in the multistep development of fibrosarcoma. *Cell* **66**: 1095–1104

Kang SM, Maeda K, Onoda N, Chung YS, Nakata B, Nishiguchi Y and Sowa M (1997) Combined analysis of p53 and vascular endothelial growth factor expression in colorectal carcinoma for determination of tumor vascularity and liver metastasis. *Int J Cancer* **74**: 502–507

Kieser A, Weich HA, Brandner G, Marme D and Kolch W (1994) Mutant p53 potentiates protein kinase C induction of vascular endothelial growth factor expression. *Oncogene* **9**: 963–969

Landriscina M, Remiddi F, Ria F, Palazzotti B, De Leo ME, Iacoangeli M, Roselli R, Scerrati M and Galeotti T (1996) The level of MnSOD is directly correlated with grade of brain tumours of neuroepithelial origin. *Br J Cancer* **74**(12): 1877–1885

Lindmark G, Gerdin B, Sundberg C, Pahlman L, Bergstrom R and Glimelius B (1996) Prognostic significance of the microvascular count in colorectal cancer. *J Clin Oncol* **14**: 461–466

Mukhopadhyay D, Tsiokas L and Sukhatme VP (1995) Wild-type p53 and v-src exert opposing influences on human vascular endothelial growth factor gene expression. *Cancer Res* **55**: 6161–6165

Smith-McCune KK and Weidner N (1994) Demonstration and characterization of the angiogenic properties of cervical dysplasia. *Cancer Res* **54**: 800–804

Takahashi Y, Kitadai Y, Bucana CD, Cleary KR and Ellis LM (1995) Expression of vascular endothelial growth factor and its receptor, KDR, correlates with vascularity, metastasis, and proliferation of human colon cancer. *Cancer Res* **55**: 3964–3968

Takahashi Y, Tucker SL, Kitadai Y, Koura AN, Bucana CD, Cleary KR and Ellis LM (1997) Vessel counts and expression of vascular endothelial growth factor as prognostic factors in node-negative colon cancer. *Arch Surg* **132**: 541–546

Tanigawa N, Amaya H, Matsumura M, Lu C, Kitaoka A, Matsuyama K and Muraoka R (1997) Tumor angiogenesis and mode of metastasis in patients with colorectal cancer. *Cancer Res* **57**(6): 1043–1046

Thomas KA (1996) Vascular endothelial growth factor, a potent and selective angiogenic agent. *J Biol Chem* **271**: 603–606

Tomisaki S, Ohno S, Ichiyoshi Y, Kuwano H, Maehara Y and Sugimachi K (1996) Microvessel quantification and its possible relation with liver metastasis in colorectal cancer. *Cancer* **77**: 1722–1728

Vermeulen PB, Verhoeven D, Fierens H, Hubens G, Goovaerts G, Van March E, De Bruijn EA, Van Oosterom AT and Dirix LY (1995) Microvessel quantification in primary colorectal carcinoma: an immunohistochemical study. *Br J Cancer* **71**: 340–343

Vlodavsky I, Bar-Shavit R, Ishai-Michaeli R, Bashkin P and Fuks Z (1991) Extracellular sequestration and release of fibroblast growth factor: a regulatory mechanism? *Trends Biochem Sci* **16**: 268–271

Warren RS, Yuan H, Matli MR, Gillett NA and Ferrara N (1995) Regulation by vascular endothelial growth factor of human colon cancer tumorigenesis in a mouse model of experimental liver metastasis. *J Clin Invest* **95**: 1789–1797

Watanabe H, Hori A, Seno M, Kozai Y, Igarashi K, Ichimori Y and Kondo K (1991) A sensitive enzyme immunoassay for human basic fibroblast growth factor. *Biochem Biophys Res Commun* **175**: 229–235

Weidner N, Semple JP, Welch WR and Folkman J (1991) Tumor angiogenesis and metastasis – correlation in invasive breast carcinoma. *N Engl J Med* **324**: 1–8

Weidner N, Folkman J, Pozza F, Bevilacqua P, Allred EN, Moore DH, Meli S and Gasparini G (1992) Tumor angiogenesis: a new significant and independent prognostic indicator in early-stage breast carcinoma. *J Natl Cancer Inst* **84**: 1875–1887

Weidner N, Carroll PR, Flax J, Blumenfeld W and Folkman J (1993) Tumor angiogenesis correlates with metastasis in invasive prostate carcinoma. *Am J Pathol* **143**: 401–409

© Cancer Research Campaign 1998


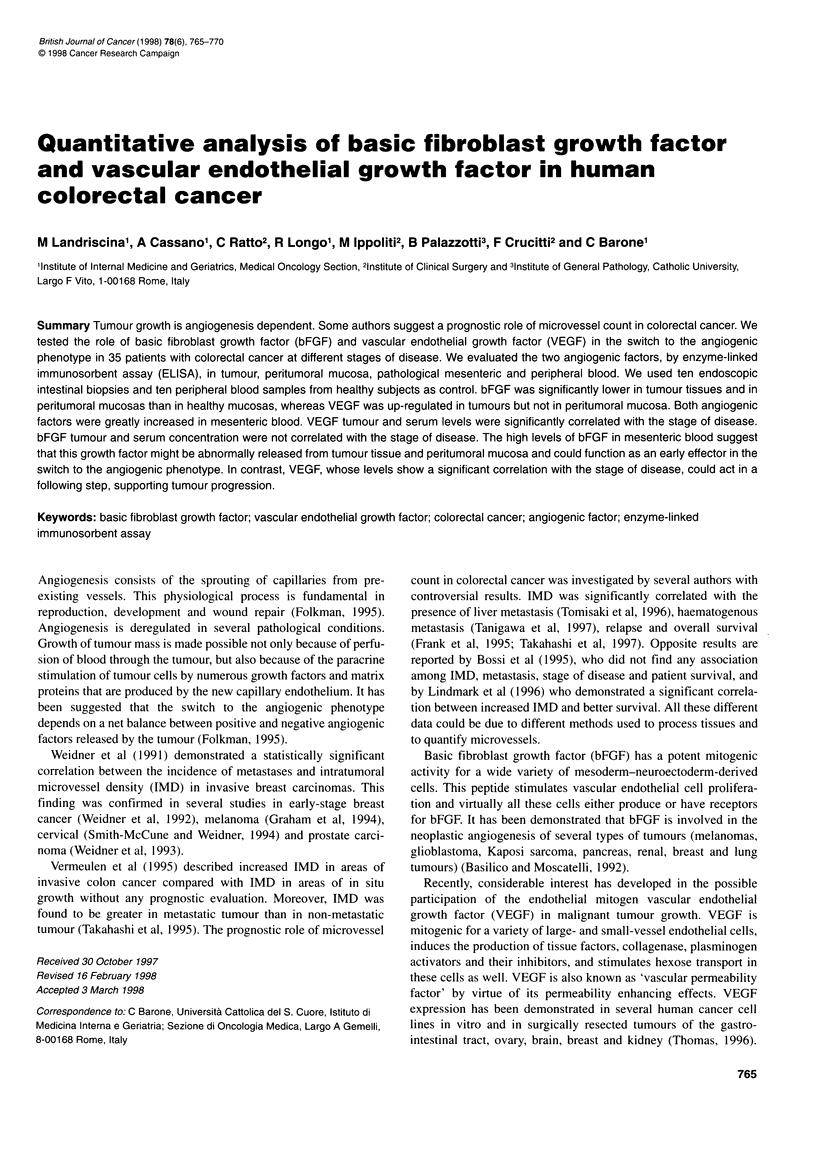

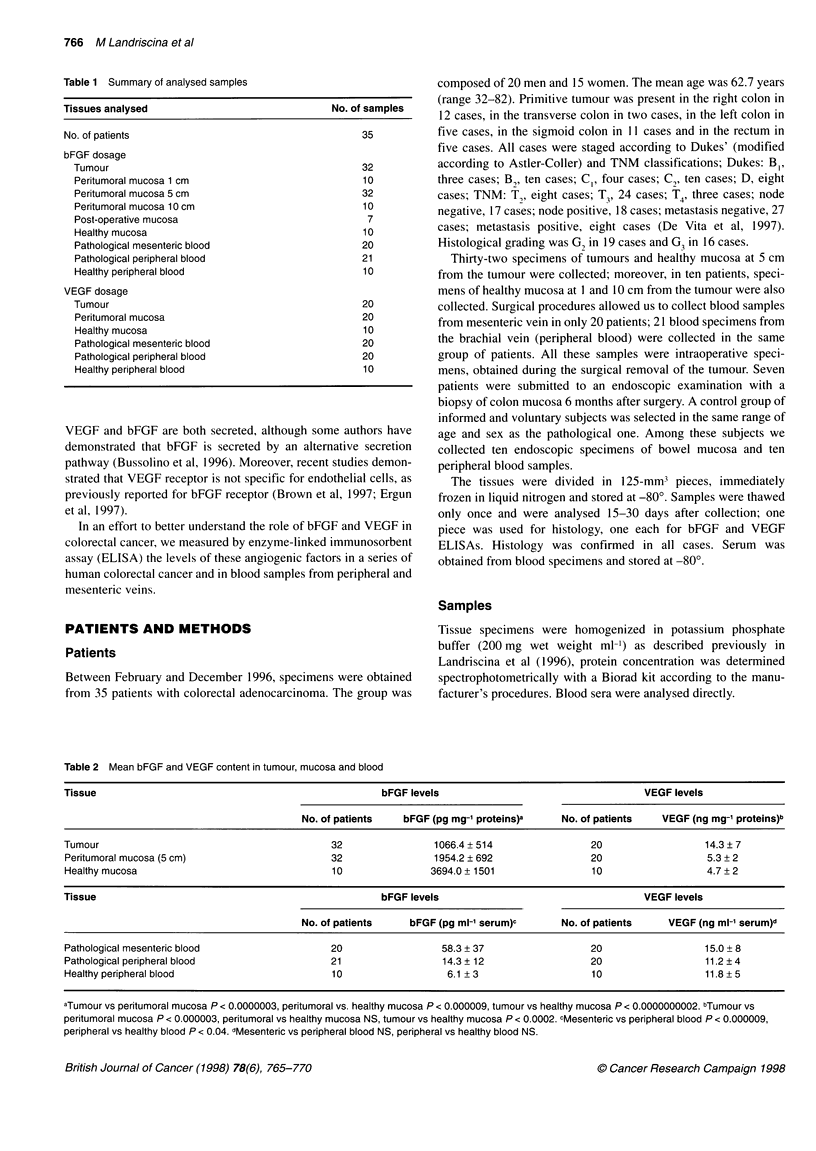

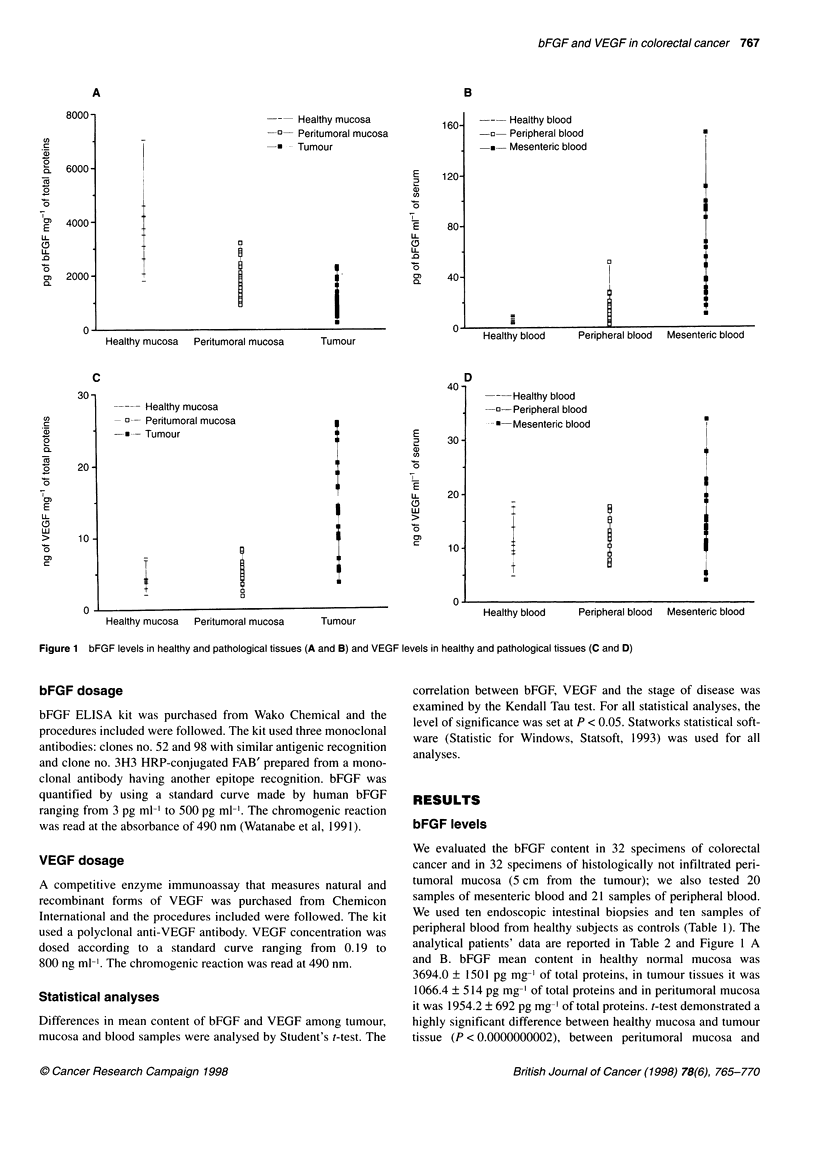

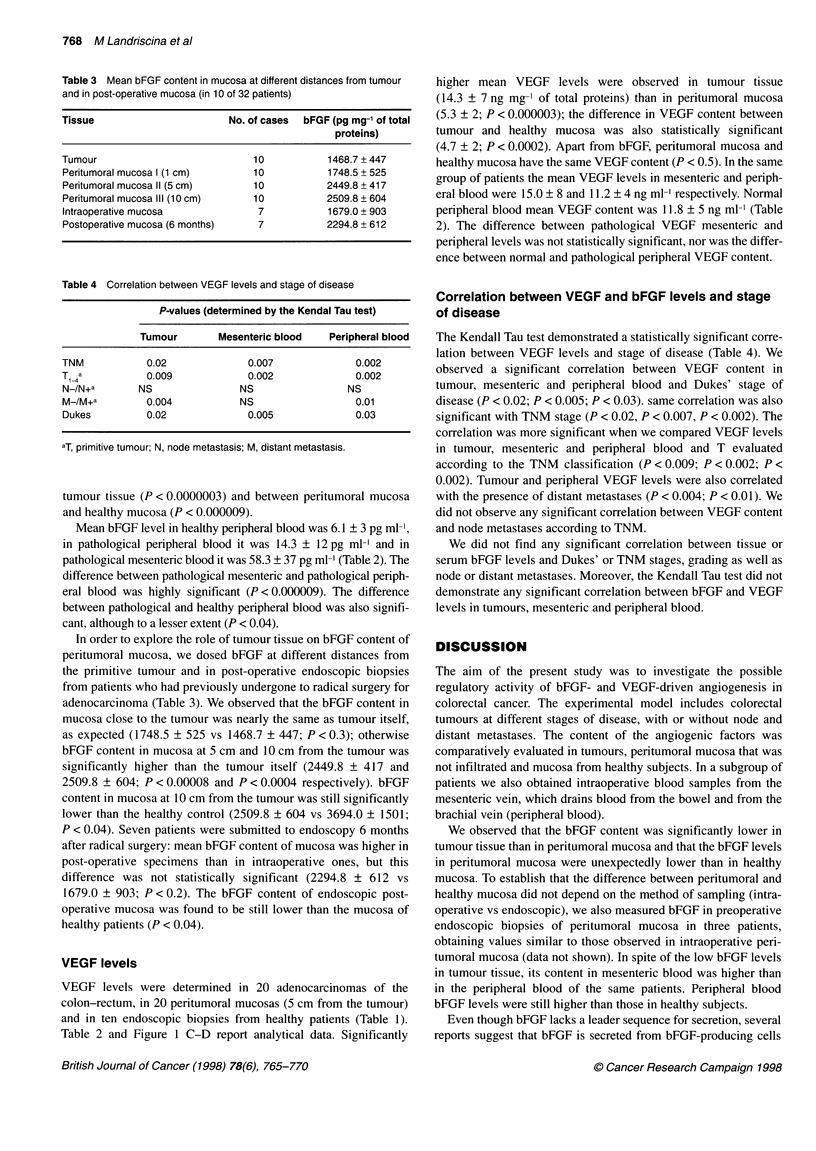

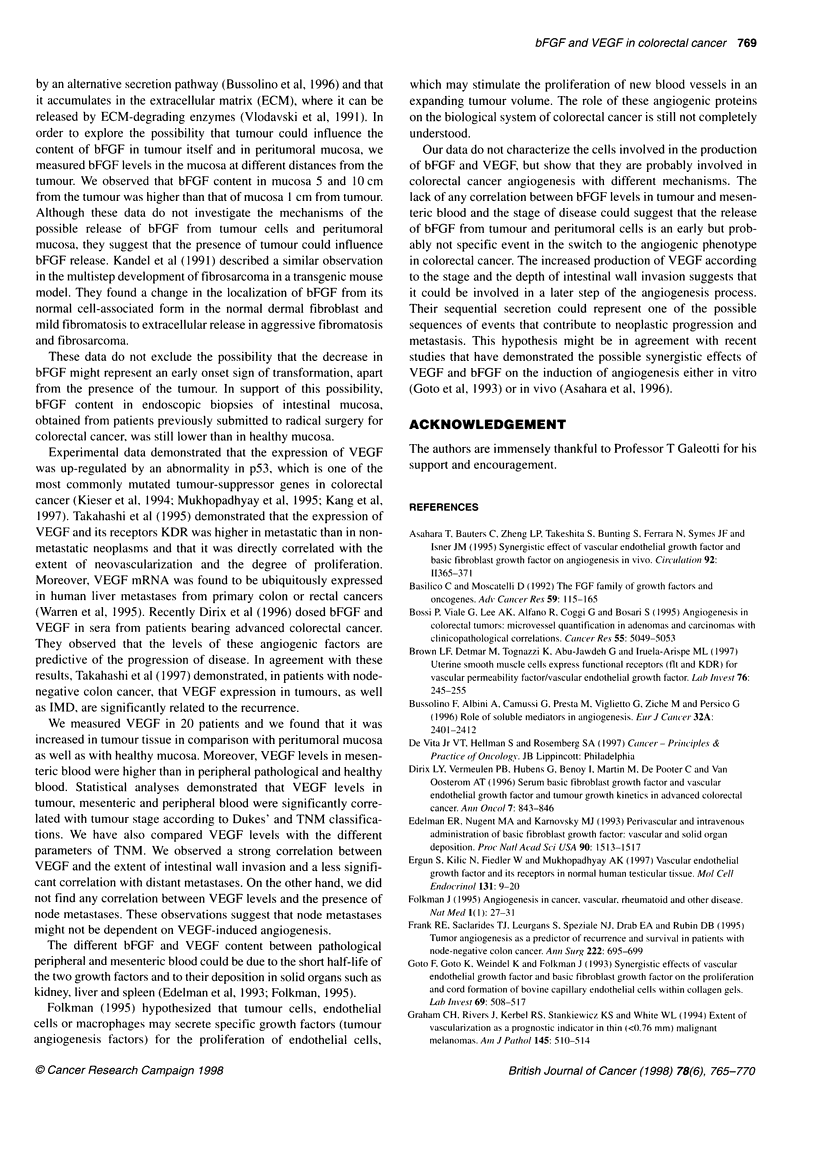

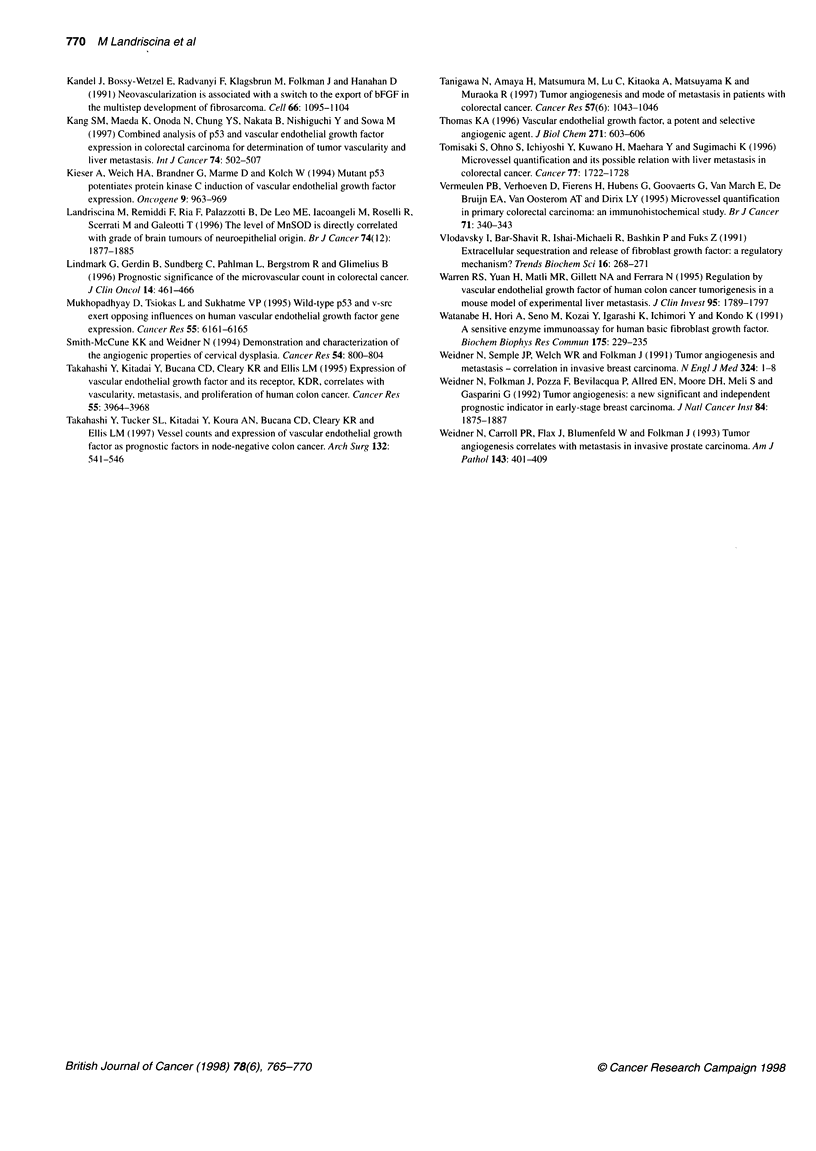


## References

[OCR_00655] Basilico C., Moscatelli D. (1992). The FGF family of growth factors and oncogenes.. Adv Cancer Res.

[OCR_00661] Bossi P., Viale G., Lee A. K., Alfano R., Coggi G., Bosari S. (1995). Angiogenesis in colorectal tumors: microvessel quantitation in adenomas and carcinomas with clinicopathological correlations.. Cancer Res.

[OCR_00664] Brown L. F., Detmar M., Tognazzi K., Abu-Jawdeh G., Iruela-Arispe M. L. (1997). Uterine smooth muscle cells express functional receptors (flt-1 and KDR) for vascular permeability factor/vascular endothelial growth factor.. Lab Invest.

[OCR_00671] Bussolino F., Albini A., Camussi G., Presta M., Viglietto G., Ziche M., Persico G. (1996). Role of soluble mediators in angiogenesis.. Eur J Cancer.

[OCR_00682] Dirix L. Y., Vermeulen P. B., Hubens G., Benoy I., Martin M., De Pooter C., Van Oosterom A. T. (1996). Serum basic fibroblast growth factor and vascular endothelial growth factor and tumour growth kinetics in advanced colorectal cancer.. Ann Oncol.

[OCR_00692] Ergün S., Kiliç N., Fiedler W., Mukhopadhyay A. K. (1997). Vascular endothelial growth factor and its receptors in normal human testicular tissue.. Mol Cell Endocrinol.

[OCR_00697] Folkman J. (1995). Angiogenesis in cancer, vascular, rheumatoid and other disease.. Nat Med.

[OCR_00701] Frank R. E., Saclarides T. J., Leurgans S., Speziale N. J., Drab E. A., Rubin D. B. (1995). Tumor angiogenesis as a predictor of recurrence and survival in patients with node-negative colon cancer.. Ann Surg.

[OCR_00706] Goto F., Goto K., Weindel K., Folkman J. (1993). Synergistic effects of vascular endothelial growth factor and basic fibroblast growth factor on the proliferation and cord formation of bovine capillary endothelial cells within collagen gels.. Lab Invest.

[OCR_00712] Graham C. H., Rivers J., Kerbel R. S., Stankiewicz K. S., White W. L. (1994). Extent of vascularization as a prognostic indicator in thin (< 0.76 mm) malignant melanomas.. Am J Pathol.

[OCR_00721] Kandel J., Bossy-Wetzel E., Radvanyi F., Klagsbrun M., Folkman J., Hanahan D. (1991). Neovascularization is associated with a switch to the export of bFGF in the multistep development of fibrosarcoma.. Cell.

[OCR_00726] Kang S. M., Maeda K., Onoda N., Chung Y. S., Nakata B., Nishiguchi Y., Sowa M. (1997). Combined analysis of p53 and vascular endothelial growth factor expression in colorectal carcinoma for determination of tumor vascularity and liver metastasis.. Int J Cancer.

[OCR_00738] Landriscina M., Remiddi F., Ria F., Palazzotti B., De Leo M. E., Iacoangeli M., Rosselli R., Scerrati M., Galeotti T. (1996). The level of MnSOD is directly correlated with grade of brain tumours of neuroepithelial origin.. Br J Cancer.

[OCR_00744] Lindmark G., Gerdin B., Sundberg C., Påhlman L., Bergström R., Glimelius B. (1996). Prognostic significance of the microvascular count in colorectal cancer.. J Clin Oncol.

[OCR_00749] Mukhopadhyay D., Tsiokas L., Sukhatme V. P. (1995). Wild-type p53 and v-Src exert opposing influences on human vascular endothelial growth factor gene expression.. Cancer Res.

[OCR_00754] Smith-McCune K. K., Weidner N. (1994). Demonstration and characterization of the angiogenic properties of cervical dysplasia.. Cancer Res.

[OCR_00758] Takahashi Y., Kitadai Y., Bucana C. D., Cleary K. R., Ellis L. M. (1995). Expression of vascular endothelial growth factor and its receptor, KDR, correlates with vascularity, metastasis, and proliferation of human colon cancer.. Cancer Res.

[OCR_00765] Takahashi Y., Tucker S. L., Kitadai Y., Koura A. N., Bucana C. D., Cleary K. R., Ellis L. M. (1997). Vessel counts and expression of vascular endothelial growth factor as prognostic factors in node-negative colon cancer.. Arch Surg.

[OCR_00773] Tanigawa N., Amaya H., Matsumura M., Lu C., Kitaoka A., Matsuyama K., Muraoka R. (1997). Tumor angiogenesis and mode of metastasis in patients with colorectal cancer.. Cancer Res.

[OCR_00787] Vermeulen P. B., Verhoeven D., Fierens H., Hubens G., Goovaerts G., Van Marck E., De Bruijn E. A., Van Oosterom A. T., Dirix L. Y. (1995). Microvessel quantification in primary colorectal carcinoma: an immunohistochemical study.. Br J Cancer.

[OCR_00795] Vlodavsky I., Bar-Shavit R., Ishai-Michaeli R., Bashkin P., Fuks Z. (1991). Extracellular sequestration and release of fibroblast growth factor: a regulatory mechanism?. Trends Biochem Sci.

[OCR_00798] Warren R. S., Yuan H., Matli M. R., Gillett N. A., Ferrara N. (1995). Regulation by vascular endothelial growth factor of human colon cancer tumorigenesis in a mouse model of experimental liver metastasis.. J Clin Invest.

[OCR_00817] Weidner N., Carroll P. R., Flax J., Blumenfeld W., Folkman J. (1993). Tumor angiogenesis correlates with metastasis in invasive prostate carcinoma.. Am J Pathol.

[OCR_00811] Weidner N., Folkman J., Pozza F., Bevilacqua P., Allred E. N., Moore D. H., Meli S., Gasparini G. (1992). Tumor angiogenesis: a new significant and independent prognostic indicator in early-stage breast carcinoma.. J Natl Cancer Inst.

